# Supplementary material for: A Direct Comparison, and Prioritisation, of the Immunotherapeutic Targets Expressed by Adult and Paediatric Acute Myeloid Leukaemia Cells: A Systematic Review and Meta-Analysis
Source: Int J Mol Sci. 2023 Jun 2;24(11):9667. doi: 10.3390/ijms24119667 (PMC10253696; doi:10.3390/ijms24119667)
Supplement: Supplementary file 1 [file ijms-24-09667-s001.zip › Morris et al SLR AML paeds and adults Supplementary Table S1 Protocol.pdf]

Aim: To conduct a systematic literature review, following PRISMA guidelines, to identify the most promising immunological targets for the treatment of adult and paediatric acute myeloid leukaemia in the literature.

Objectives: The systematic search will look to identify tumour associated antigens and their expression levels for use as immunotherapeutic targets.

'Promising targets' will be defined as those who are:

- Expressed consistently throughout both patient populations
- Expressed at high enough levels to allow for targeted drug application
- Minimal/no expression within healthy tissues to ensure anti-cancer therapy has minimal effect on healthy cells whilst a high drug payload is still delivered to diseased cells

Collated data will be examined through meta-analysis via STRING and PANTHER. Identified antigen(s) will be examined for their use in immunotherapy through their function in biological pathways and percentage expression both within their analyses and in existing literature.

Search Criteria:

Human models for both adult and paediatric AML will be considered. The meta-analysis carried out will be conducted separately for adult and paediatric AML, before results are critically compared.

	Inclusion	Exclusion
Study Design and Parameters	• Acute Myeloid Leukemia<br>• Human Model<br>• All Age Groups<br>• M4/M5 subgroups	• Other cancer types including Chronic Myeloid Leukemia<br>• Animal Models<br>• Cell-line Studies<br>• Other subgroups
Sources	• Primary Literature<br>• Reviews<br>• Clinical Trials<br>• English Language	• Conferences<br>• Letters to editors<br>• Case Reports<br>• Texts that cannot be translated into English

	• Over 10 Participants<br>• All geographical locations of participants	

Search methodology:

The following search terms with the included Boolean operators will be used to search titles and abstracts in all databases: "acute myeloid leukaemia" OR "acute myeloid leukemia" OR "acute myelogenous leukaemia" OR "acute myelogenous leukemia" AND antigen OR “antigen therapy” AND human

The following databases will be searched:

- PubMed
- MEDLINE
- Scopus
- Cochrane Library
- CINAHL

Data collection and analysis

Once searches are conducted results will be exported to EndNote so that duplicates are removed. Next the remaining papers were exported to Excel and titles are screened for relevance and excluded based on the criteria detailed above. The abstracts of the remaining papers will be read and excluded in the same way. After this, the full text of the remaining articles will be read and any that do not meet the inclusion criteria will be removed.

For the final included studies, the following information will be extracted:

- Study Type
- Sample Size
- AML Subtype
- Antigen(s) under investigation
- Expression levels of antigen(s)
- Brief Methodology

- Outcomes
